# Ddx56 maintains proliferation of mouse embryonic stem cells via ribosome assembly and interaction with the Oct4/Sox2 complex

**DOI:** 10.1186/s13287-020-01800-w

**Published:** 2020-07-23

**Authors:** Jingwen Wang, Jiahui Liu, Miaoman Ye, Feng Liu, Su Wu, Junjiu Huang, Guang Shi

**Affiliations:** 1 MOE Key Laboratory of Gene Function and Regulation, State Key Laboratory of Biocontrol, School of Life Sciences, Sun Yat-sen University, Guangzhou, 510275 China; 2 Key Laboratory of Reproductive Medicine of Guangdong Province, The First Affiliated Hospital and School of Life Sciences, Sun Yat-sen University, Guangzhou, 510275 China

**Keywords:** Embryonic stem cells, Ddx56, Proliferation, Ribosome assembly, Oct4/Sox2 complex, Cell cycle

## Abstract

**Background:**

Embryonic stem cells (ESCs) are important source of clinical stem cells for therapy, so dissecting the functional gene regulatory network involved in their self-renewal and proliferation is an urgent task. We previously reported that Ddx56 interacts with the core transcriptional factor Oct4 by mass spectrometry analysis in ESCs. However, the exact function of Ddx56 in ESCs remains unclear.

**Methods:**

We investigated the role of Ddx56 in mouse ESCs (mESCs) through both gain- and loss-of-function strategies. The effect of Ddx56 on mESCs was determined based on morphological changes, involvement in the network of pluripotency markers (Nanog, Oct4, Sox2), and altered lineage marker expression. In addition, the role of Ddx56 in mESCs was evaluated by polysome fractionation, qRT-PCR, and co-immunoprecipitation (co-IP). Finally, RNA sequencing was applied to explore potential network regulation by Ddx56 in mESCs.

**Result:**

We found that Ddx56 participated in ribosome assembly, as knockout or RNAi knockdown of Ddx56 led to ribosome dysfunction and cell lethality. Surprisingly, exogenous expression of C-terminal domain truncated Ddx56 (Ddx56 ΔC-ter) did not affect ribosome assembly, but decreased mESC proliferation by downregulation of proliferation-related genes and cell cycle changing. In terms of mechanism, Ddx56 interacted with the Oct4 and Sox2 complex by binding to Sox2, whereas Ddx56 ΔC-ter showed weaker interaction with Sox2 and led to retardation of mESC proliferation.

**Conclusions:**

Ddx56 maintains ESC proliferation by conventional regulation of ribosome assembly and interaction with the Oct4 and Sox2 complex.

## Introduction

Embryonic stem cells (ESCs) originate from the inner cell mass of mammalian blastocysts [1, 2]. Under appropriate culture conditions, undifferentiated ESCs proliferate infinitely with the potential to differentiate into all three germ layers, including endoderm, mesoderm, and ectoderm [3]. As a cell model for the study of early embryo development, ESCs are promising in the application of regenerative medicine [4–6].

Oct4, Sox2, and Nanog are three of the major pluripotent transcription factors which play crucial roles in the maintenance of ESC properties [7]. By immunoprecipitation (IP) and mass spectrometry (IP-MS) analysis, many new pluripotency-associated factors were discovered [8]. The finding of these factors contribute to a better understanding of the molecular mechanisms underlying self-renewal, proliferation, and pluripotency maintenance in ESCs, which is quite important to fulfill our knowledge about the potential of ESCs.

Nucleoli is a sub-nuclear region where ribosome assembly takes place. In ESCs, the nucleoli undergo dynamic morphological changes during the differentiation process [9, 10]. In addition, global mRNA translational levels are also altered in the heterogeneous mESCs [11, 12]. Most of the nucleolus proteins are essential [13], and some nucleolus proteins, like nucleolin or fibrillarin, are highly expressed in mESCs, regulating early development and mESC identity [14–16]. Knocking down of these genes affects ribosomal DNA transcription or ribosome biogenesis, leading to decreased cell proliferation and stem cell differentiation [16–19]. Therefore, nucleolus proteins are important in ESC self-renewal and proliferation.

In our previous IP-MS analysis, nucleolus proteins nucleophosmin 1 (Npm1) and DEAD-box helicase 56 (Ddx56) were potential interactors of Oct4. Ddx56 belongs to the RNA helicase family. Genes of this family are widely expressed and participate in all aspects of RNA metabolism, including transcription, pre-mRNA splicing, ribosome biogenesis, translation, and mRNA decay [20–22]. In addition, RNA helicases also act on stem cell pluripotency or differentiation [23]. As a family member, Ddx56 is indispensable for the assembly of the West Nile virus in a RNA helicase activity-dependent manner [24–26]. Ddx56 is also identified as a constituent of free nucleoplasmic 65S preribosomal particles, suggesting it might play a role in the assembly of the large (60S) ribosomal subunit [27]. However, the function of Ddx56 in the regulation of ribosome assembly and its specific role in ESCs are not fully understood.

In this study, either Ddx56 knockout or knockdown in mouse ESCs (mESCs) led to ribosome assembly defects and cell lethality, whereas inducible overexpression of wildtype (WT) did not. These results suggested Ddx56 was essential for the ribosome assembly of mESCs. To explore its ribosome-independent function in mESCs, we overexpressed different Ddx56 truncated mutants in mESC lines. The expression of C-terminal domain truncated Ddx56 (Ddx56 ΔC-ter) in mESCs led to the formation of notably smaller clones without affecting ribosome assembly. Further experiments showed that Ddx56 was an essential protein for mESC proliferation, which is dependent on its interaction with the Oct4/Sox2 complex.

## Materials and methods

### Cell lines and cell culture

The mouse ESC lines used were E14 and A17-2loxP mESCs (gift from Dr. Thomas P. Zwaka). Mouse ESCs were maintained under feeder-free conditions, on culture dishes coated with 0.1% gelatin (Sigma-Aldrich). Cells were cultured in Knockout DMEM (Gibco) supplemented with 15% (v/v) fetal bovine serum (Hyclone, Australia), 0.1 mM β-mercaptoethanol (Sigma), 2 mM GlutaMAX, 0.1 mM minimum essential medium (MEM) non-essential amino acids (Sigma), 100 U/mL penicillin (Gibco), 100 μg/mL streptomycin (Gibco), 1000 U/mL LIF (Millipore), 1 μM PD0325901 (Stemgent), and 3 μM CHIR99021 (Stemgent). 293T cells were from ATCC company (America) and cultured in DMEM (Corning) supplemented with 10% (vol/vol) FBS (Excell, Australia), 100 U/mL penicillin (Gibco), and 100 μg/mL streptomycin (Gibco).

### Stable cell line generation

Sequence encoding *Cas9*, murine *Oct4*, *Sox2*, *Ddx56*, and *EGFP* were cloned into pENTR. *Ddx56* domain truncations were generated by overlap PCR on pENTR-*Ddx56*. *Cas9*, *Oct4*, *Sox2*, *EGFP*, *Ddx56*, and its domain truncations were ligased to pDEST27 or pBabe by gateway® LR clonase reaction (Thermo Fisher) for immunoprecipitation experiments. A17-2loxP mESCs have a doxycycline-inducible promoter and two loxP loci near *HPRT* gene. *Cas9*, *Ddx56*, and its domain truncations were ligated to p2loxP plasmid, then cotransfected with pSalk-Cre plasmid which codes cyclization recombination enzyme (Cre) into A17-2loxP mESCs by Lipofectamine 2000 (Invitrogen). Positive ESC clones were selected by G418 and detected by western blot after adding doxycycline for 48 h. A pair of gRNA oligonucleotides with 5′-CACC and 3′-AAAC overhang was synthesized, annealed, and ligased to vectors (px330, px458, plenti-gRNA1-BSD, and plenti-gRNA2-Hygro). To get inducible knockout *Ddx56* cell lines, plenti-gRNA2-BSD and plenti-gRNA2-Hygro were transfected into A17-2loxP-Cas9 cells via retroviral transfection system. The *Ddx56* gRNA sequences were as follows: gRNA1, GCCATTCCTCTGGCGCTGG; gRNA2, GTGGTCTGTGAGACAGAAG. Target sites of *Ddx56* were PCR amplified using primers in Additional file 1: Table S1. The PCR products were then used in T7 endonuclease I (T7EI) cleavage assay.

### siRNA transfection

E14 cells were transfected with siRNA oligos targeting Ddx56 using RNAi Max (Invitrogen) and harvested 48 h after transfection. The small interfering RNA (siRNA) oligos were purchased from Guangzhou  IGE Biotechnology Ltd., and their sequences are listed below:

- *Ddx56*-si-1, CAGAGAAGCUCAAGACAUA
- *Ddx56*-si-2, UUUAGGAUCCAGUCUUUCC
- *Ddx56*-si-3, GAGCUGUGUUGAUGGAGAA

### Quantitative real-time PCR (qRT-PCR)

Total RNA was isolated using phenol-chloroform. The following cDNA synthesis was performed with PrimeScript™ RT Reagent Kit (TaKaRa), and qRT-PCR using GoTaq® qPCR Master Mix (Promega) was performed using qPCR primers with ABI StepOnePlus Real-Time PCR System. The primers are listed in Additional file 1: Table S1.

### Co-immunoprecipitation (co-IP) and western blotting

293T cells were transfected with various expression vectors and harvested 48 h after transfection. Cells were lysed in 1× NETN buffer (40 mM Tris-HCl (pH 8.0), 100 mM NaCl, 0.5% NP40, 1 mM EDTA, 10% glycerol, containing protease inhibitors). The supernatant was incubated with Glutathione Sepharose 4B for 4 h at 4 °C. The immunoprecipitates were then eluted by SDS loading buffer. Whole cell lysates were also obtained by direct lysis of cells in SDS loading buffer. All samples were resolved by SDS-PAGE and transferred onto nitrocellulose filter membrane (Millipore) for blotting with appropriate antibodies. Antibodies for western blotting are anti-Flag (mouse) (Abmart M20008M), anti-GAPDH (mouse) (Proteintech group 60004-1-Ig), anti-GST (Rabbit) (Homemade), anti-mouse 680 (LI-COR 926-32220), and anti-rabbit 800 (LI-COR 926-32211).

### His-tagged recombinant protein purification and pulldown in vitro

Sox2-His plasmid was transformed into *E. coli* strain BL21, and the fusion protein expression was induced by adding isopropyl thio-β-d-galactosidase (IPTG) in 1 mM final concentration at 18 °C. After 18 h, cells were centrifuged for 10 min at 4000*g* and 4 °C. The cell pellets were re-suspended in lysis buffer (Tris 50 mM, 500 mM NaCl, 10% glycerol, 0.5% NP40, 1 mM DTT, 1 mM EDTA, 1 mM PMSF, and protease inhibitor cocktail) and lysed with a sonicator. Cell lysis was centrifuged at maximum speed in a microcentrifuge for 10 min at 4 °C, then the supernatant was transferred to Ni-NTA resin column with incubation for 30 min. The column was washed for three times, then the His-tagged Sox2 affinity beads were detected by SDS-PAGE. 293T cells were collected in 48 h after transfected with *Ddx56* full length and ΔC-ter plasmids, and grayscale in western blot experiment was used to balance the quantity of protein. The same quality of protein was added into beads and incubated 4 h at 4 °C with gentle agitation. The beads were washed and used for western blotting. Antibodies for western blotting are anti-GST (rabbit) (Homemade), anti-Sox2 (mouse), anti-mouse 680 (LI-COR 926-32220), and anti-rabbit 800 (LI-COR 926-32211).

### Polysome fractionation

A17-2loxP mESCs were cultured in 60-mm dish and have been ~ 80% confluent on the day of the experiments. Firstly, the cells were treated with cycloheximide at a final concentration of 100 μg/mL in culture media for 5 min at 37 °C and washed once with 5 mL of ice-cold 1× PBS containing 100 μg/mL cycloheximide. Secondly, the cells were lysed with lysis buffer (140 mM NaCl, 5 mM MgCl_2_, 10 mM Tris-HCl pH 8.0, 1% Triton X-100, 0.5% sodium deoxycholate, 0.4 U/μL RNase inhibitor, 20 mM DTT, 0.1 mg/mL cycloheximide, 10 mM RVC, 0.1% cocktail), and incubated on ice for 15 min. Then, cell lysate was centrifuged at maximum speed (> 13,000 rcf) at 4 °C for 5 min. At last, the lysate supernatant was carefully transferred to the linear 10 to 50% sucrose gradients and centrifuged at 36,000 rpm for 2 h at 4 °C using the SW41Ti rotor. The sample was analyzed with a fraction collector and UV detector.

### Propidium iodide (PI) staining

After doxycycline treatment for 3 days, the cells were collected and seeded on a 15-mm microscope cover glasses for 12 h. The cells on glasses were fixed, washed twice with cold PBS, and were treated with PI and Hoechst for 15 min at dark place. Then, the cells on glasses were detected using a fluorescence microscope.

### Annexin V-FITC and PI staining

Cells were treated with or without Dox for 3 days, collected by centrifugation at 1200 rpm for 5 min, and washed twice with cold 1× PBS. Annexin V (5 μL) and PI (5 μL) were added and mixed gently and incubated in room temperature and dark place for 15 min. Then, the cells were analyzed by fluorescence-activated cell sorting (FACS) (Kit: Invitrogen Catalog no. V13241).

### Cell cycle assay

After doxycycline treatment for 6 days, the cells were collected by centrifugation at 1200 rpm for 3 min, and washed once with 1× PBS. The cells were resuspended with 70% ethanol and stored at 4 °C overnight. The cells were washed twice with 1× PBS in the second day, resuspended with 200 μL 1× PBS containing 2 μL RNase in 37 °C for 30 min, and incubated with PI at a final concentration of 10 μg/mL for 5 min on ice. Then, the cells were analyzed by fluorescence-activated cell sorting (FACS) to determine cell cycle stages.

### Immunofluorescence

Cells were seeded on 15-mm microscope cover glasses a day before the experiment. For immunostaining, cells were fixed with 4% paraformaldehyde, permeabilized (5% Triton-X, 20 mM HEPES, 3 mM MgCl_2_·6H_2_O, 300 mM sucrose), blocked (3% goat serum, New Zealand origin (16210072, Gibco, Thermo Fisher), 0.1% BSA in PBS), and incubated with primary antibodies overnight at 4 °C. Then, they were stained with Alexa Fluor-conjugated secondary antibodies goat anti-rabbit IgG (H+L) (Thermo Fisher) and goat anti-mouse IgG (H+L) (Thermo Fisher) at room temperature for 1 h. Nuclear staining was performed with DAPI. Antibodies for immunofluorescence are anti-Flag (rabbit) (GenScript A00170-40) and anti-Oct4 (mouse) (BD 611202).

### Cell proliferation

A17-2loxP-Ddx56, Ddx56ΔC-ter, and GFP cells were cultured in a 24-well plate; each well contains 4000 cells. After 24 h, cells were treated with or without doxycycline. And cells were counted at 2, 4, and 6 days. Each type has three wells repeats.

### RNA sequencing and analysis

A17-2loxP-Ddx56, Ddx56ΔC-ter, and GFP cells were cultured and treated with doxycycline for 48 h. The cells were harvested and dissolved in TRIzol for total RNA extraction and treated with DNase I (Ambion) to remove any potential contaminated DNA fraction. The following library generation and sequencing were conducted by Ruibo Biotechnology Co., Ltd. in Guangzhou. The RNA-seq reads were mapped to the UCSC mouse genes (mm10). Only the uniquely mapped reads were retained for analysis in our study. Fragment per kilobase per million mapped fragments (FPKM) and DE genes were calculated.

### Statistical analysis

Significant differences between groups were calculated by performing a two-way ANOVA and were defined as *p < 0.05, **p < 0.01, and ***p < 0.001. The error bars represent standard error of the mean (SEM) of three independent experiments.

## Results

### Ddx56 knockout leads to mESC lethality

**Fig. 1:**
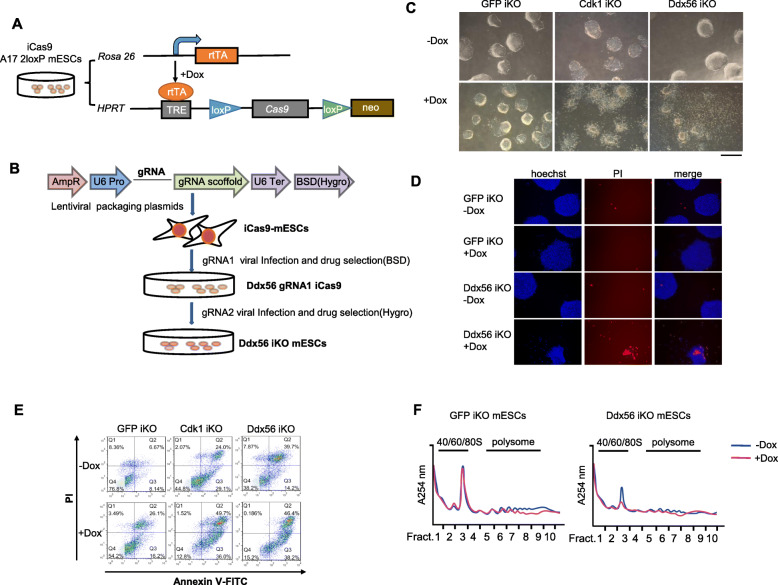
Ddx56 knockout affects ribosome assembly and leads to cell lethality. **a** The generation of doxycycline (Dox)-inducible Cas9 expression in A17-2loxP mESCs (iCas9 mESCs). **b** The strategy of generating doxycycline-inducible Ddx56 knockout ESC line (Ddx56 iKO mESCs). Bar, 600 μm. **c** Ddx56 iKO mESCs were cultured with doxycycline (1 μg/mL) (+Dox) or without doxycycline (−Dox) for 3 days. GFP iKO mESCs served as a negative control. Cdk1 iKO mESCs served as a positive control. Cells were visualized by phase-contrast microscopy. × 4, objective. **d** Cells were stained with propidium iodide (PI) and then observed under a fluorescence microscope. **e** Cells were stained with PI and Annexin V-FITC, subjected to FACS analysis. **f** Cells were cultured with (+Dox) or without (−Dox) doxycycline for 2 days and then subjected to ribosome profiling

To investigate the role of Ddx56 in mESCs, we first generated doxycycline (Dox)-inducible Cas9 expression in A17-2loxP mESCs (iCas9 mESCs) as reported previously [28]. A17-2loxP mESC line carried the Tet-on system and the Cre/loxP recombination system. rtTA was inserted into the *Rosa26* locus and the exchange cassette flanked by heterologous, self-incompatible loxP sites was designed downstream of a doxycycline (tetracycline)-responsive promoter (TRE) in the *HPRT* locus containing *Cas9* gene. The iCas9 mESC line was generated by drug selection (Fig. 1a). Two gRNAs were designed targeting both the Ddx56 helicase ATP binding domain and C-terminal domain individually (Figure S1A). They were transfected into E14 mESCs to test the cleavage efficiency by T7 endonuclease 1 (T7E1) assay, and the results indicated both gRNAs could efficiently target the desired sites (Figure S1B). The two guide RNAs (gRNAs) were then cotransferred into the iCas9 mESCs through lentivirus carrying different drug resistance (Fig. 1b). Dox treatment induced Cas9 expression and finally generated the Ddx56 inducible knockout mESCs (iKO mESCs) by two gRNAs (Fig. 1c).

Cell cycle regulator Cdk1 is essential for cell growth [29]. We also established the Cdk1 iKO mESCs by infecting the iCas9 mESCs with a Cdk1 targeting gRNA. After treatment with Dox for 3 days, we found that Cdk1 iKO mESCs and Ddx56 iKO mESCs could not form normal colonies (Fig. 1c). Through propidium iodide (PI) staining, more PI-positive cells were observed in Ddx56 iKO mESCs (Fig. 1d). PI and Annexin V staining by flow cytometry were carried out. A higher percentage of Ddx56 iKO cells underwent apoptosis compared with the GFP iKO mESCs (46.4% vs. 26.1%, Fig. 1e).

It has been reported that Ddx56 exists in the 65S preribosomal particles [27]. Polysome fractionation was performed to determine whether Ddx56 iKO impaired ribosome assembly. The absorption peak of 60S ribosome subunit and 80S mature ribosome at 260 nm in Ddx56 iKO mESCs sharply decreased compared with GFP iKO mESCs (Fig. 1f). Therefore, cell lethality in Ddx56 iKO mESCs had been observed due to ribosome defection, indicating *Ddx56* may be an essential gene.

To further verify these data, transient knockdown *Ddx56* in mESCs was carried out using three small interfering RNAs (siRNAs). Two siRNAs were able to effectively reduce *Ddx56* levels (> 50%) (Figure S2A). Ddx56 knockdown also induced cell lethality (Figure S2B). We then performed qRT-PCR to examine the mRNA expression level of pluripotent markers including *Oct4*, *Nanog*, and *Sox2*, and various lineage markers including primitive endoderm (*Gata4*, *Gata6*, *Sox7*), endoderm (*Sox17*, *Foxa2*), mesoderm (*Brachyury*, *Actc1*), and ectoderm (*Mash1*, *Nestin*). However, the expression of these markers showed no obvious changes after Ddx56 knockdown, except for *Gata6* and *Mash1* (Figure S2C). Complete disruption of Ddx56 function quickly led to cell death, which might explain why levels of the pluripotent markers did not change in a short time.

### Ddx56 ΔC-ter overexpression attenuates mESC proliferation

**Fig. 2:**
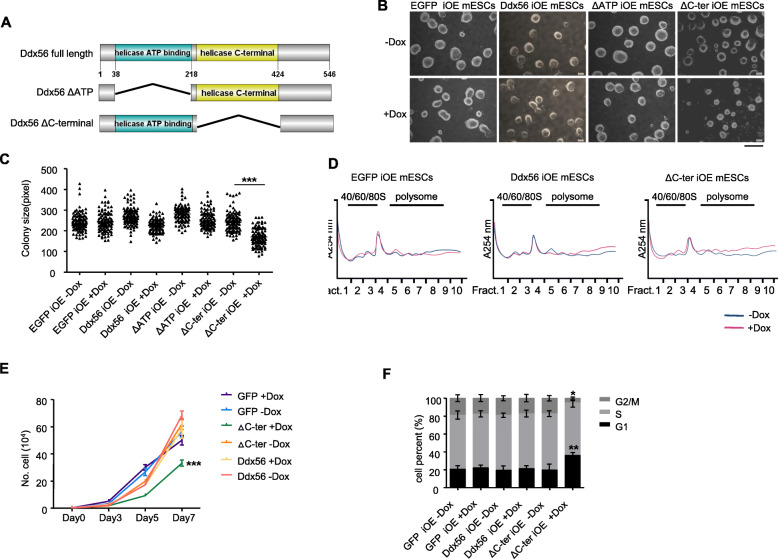
Ddx56 ΔC-ter overexpression impairs mESC proliferation. **a** Schematic diagram of full-length Ddx56 and various domain truncations. **b** mESCs were cultured with Dox (1 μg/mL) or without Dox for 4 days and visualized by phase-contrast microscopy. EGFP iOE mESCs served as a negative control. × 4, objective. Bar, 800 μm. **c** Colony size from **b** was quantified by pixels per inch (PPI), 100 colonies were calculated. Error bars indicate SD (*n* = 3), ***p < 0.001. **d** Cells were cultured with (+Dox) or without (−Dox) doxycycline for 2 days, then subjected to polysome fractionation for ribosome**. e** Cells were treated with doxycycline 24 h after seeding and were counted after 2, 4, and 6 days. Error bars indicate SD (*n* = 3), ***p < 0.001. **f** Cell cycle was measured by flow cytometry with propidium iodide staining (PI) after treatment with doxycycline for 6 days. Error bars indicate SD (*n* = 3), *p < 0.05, **p < 0.01

To explore whether Ddx56 affected mESC proliferation, we generated Dox-inducible expression of 3× Flag-tagged Ddx56 in A17-2loxP mESCs (Ddx56 iOE mESCs) similar to iCas9 construction strategy as shown in Fig. 1a. The mouse Ddx56 protein has two conserved domains, helicase ATP binding domain and helicase C-terminal domain. We then constructed two domain truncations and established their inducible overexpression in cell lines (Ddx56 ΔATP iOE mESCs and Ddx56 ΔC-ter iOE mESCs) (Fig. 2a). As indicated by western blotting, full-length Ddx56, helicase ATP binding domain deletion of Ddx56 (Ddx56 ΔATP), and helicase C-terminal domain deletion of Ddx56 (Ddx56 ΔC-ter) were inducibly expressed in the Dox-treated ESCs (Figure S3A).

Next, we performed a colony formation assay using Ddx56 full length and truncations overexpressing cells. After treatment with Dox for 4 days, we found that cells with Ddx56 ΔC-ter overexpression resulted in smaller cell colonies, while Ddx56 full length and Ddx56 ΔATP overexpression formed normal colonies (Fig. 2b). We then further quantified colony size by measuring the average colony diameter. As shown in Fig. 2c, the average colony size in Ddx56 ΔC-ter treated with Dox was significantly smaller than that in any other group, suggesting Ddx56 ΔC-ter expression might affect ribosome function, mESC proliferation, or cell size. Further analysis showed that Ddx56 ΔC-ter expression did not affect ribosome polysome fractionation and cell size (Fig. 2d, S3B), and Ddx56 ΔC-ter mESCs treated with Dox showed decreased cell proliferation compared with Ddx56 full length expressing mESCs (Fig. 2e). According to the cell cycle profiling in mESCs, Ddx56 ΔC-ter mESCs treated with Dox showed an obvious increase in G1 phase populations and a concomitant decrease in S and G2/M phase populations, suggesting that overexpression of Ddx56 ΔC-ter led to cell cycle change (Fig. 2f). These data indicated that the smaller size of mESC clonies by Ddx56 ΔC-ter overexpression was partially mediated by cell cycle perturbation.

### Ddx56 ΔC-ter overexpression affects cell cycle genes

**Fig. 3:**
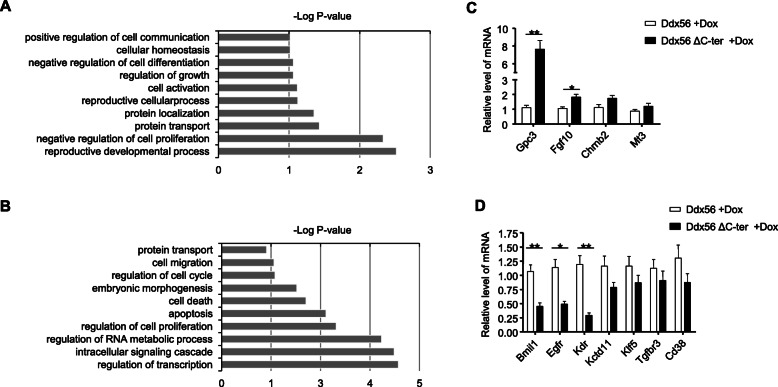
Ddx56 ΔC-ter overexpression upregulates cell cycle genes. **a**, **b** Gene Ontology analyzed upregulated (**a**) or downregulated (**b**) genes in Ddx56 ΔC-ter overexpression mESCs. **c**, **d** The selected genes were analyzed by qRT-PCR. Error bars indicate SD (*n* = 3), *p < 0.05, **p < 0.01

Given that Ddx56 ΔC-ter overexpression impaired mESC proliferation, not ribosome assembly, exploring the ribosome-independent function of Ddx56 in ESCs was necessary. We firstly detected the pluripotent genes and cell cycle genes by RT-PCR. There were no significant changes in some of these pluripotent genes, such as *Oct4*, *Sox2*, *Nanog*, *Klf4*, and *Foxd3* (Figure S4). To further explore the global transcriptome changes induced by Ddx56 ΔC-ter overexpression, we performed RNA sequencing analysis of Ddx56 iOE mESCs and Ddx56 ΔC-ter iOE mESCs treated with Dox for 2 days. Applying a cutoff threshold of fold change 2 (reads > 20), 190 genes were upregulated and 682 genes were downregulated in Ddx56 ΔC-ter overexpression mESCs compared to Ddx56 iOE mESCs. We then performed a Gene Ontology (GO) analysis of these differentially expressed genes (DEGs). The upregulated DEGs were related to positive regulation of cell communication and negative regulation of cell proliferation and cell activation (Fig. 3a). Downregulated DEGs were related to the regulation of cell cycle, regulation of cell proliferation, and regulation of transcription (Fig. 3b). We further confirmed genes involved in negative regulation of cell proliferation in upregulated DEGs (*Gpc3*, *Fgf10*, *Chrnb2*, *Mt3*) (Fig. 3c) and genes involved in the regulation of cell proliferation in downregulated DEGs (*Bmi1*, *Egfr*, *Kdr*, *Kctd11*, *Klf5*, *Tgfbr3*, *Cd38*) (Fig. 3d) by qRT-PCR assay.

### Ddx56 interacts with Sox2

**Fig. 4:**
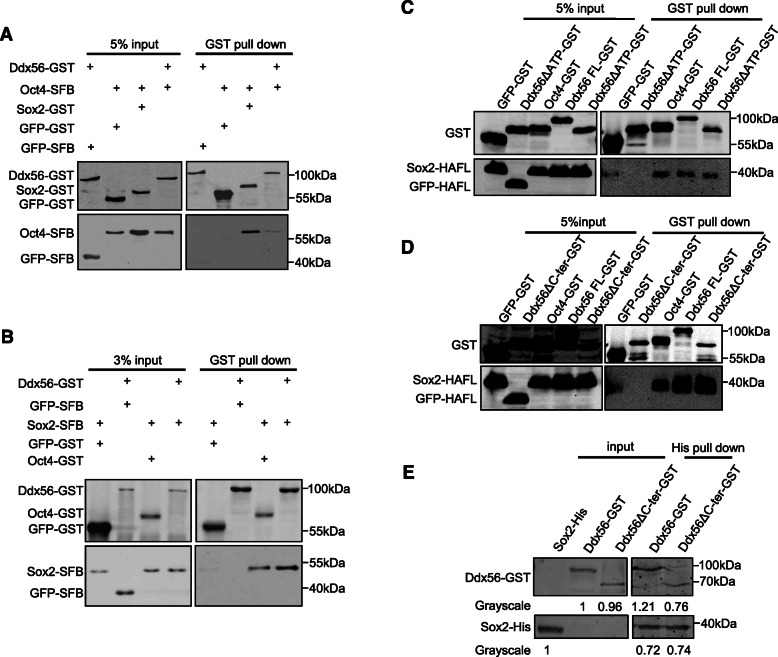
Ddx56 interacts with Oct4 and Sox2. **a** Ddx56-GST and Oct4-SFB plasmids were cotransfected into 293T cells, followed by IP and western blotting experiments. GST beads were used for pulldown. Negative controls: Ddx56-GST+GFP-SFB and GFP-GST+Oct4-SFB. Positive control: Sox2-GST+Oct4-SFB. **b** Ddx56-GST and Sox2-SFB plasmids were cotransfected into 293T cells, followed by IP and western blotting experiments. GST beads were used for pulldown. Negative controls: Ddx56-GST+GFP-SFB and GFP-GST+Sox2-SFB. Positive control: Oct4-GST+Sox2-SFB. **c**, **d** GST-tagged Ddx56 truncations and Sox2-HA plasmids were cotransfected into 293T cells, followed by IP and western blotting. **e** GST-tagged Ddx56 and Ddx56 ΔC-ter plasmids were transfected into 293T cells separately and measured by western blot. His-Sox2 protein was purified by Ni-sepharose purification. Using the same quantity of GST-Ddx56 and Ddx56 ΔC-ter separately, pulldown was performed to detect interaction

Considering that Ddx56 was found in Oct4-related protein in mESCs by mass spectrometry, we then tried to confirm the interaction between Ddx56 and Oct4. We overexpressed Flag-Ddx56 in mESCs and performed immunofluorescence staining. We found that Ddx56 could co-localize with Oct4 in the nucleus (Figure S5). Then, we examined the interaction between Ddx56 and Oct4 by GST pulldown. The result indicated that Ddx56 could interact with Oct4, but their interaction was much weaker than the interaction between Sox2 and Oct4 (Fig. 4a). In ESCs, it is well known that Oct4 and Sox2 heterodimer regulates many key pluripotent-related genes, then we examined the interaction between Ddx56 and Sox2. We found that Ddx56 also interacted with Sox2, which was as strong as the Oct4 and Sox2 heterodimer (Fig. 4b). These data suggested Ddx56 might mainly interact with Sox2. Next, we sought to determine which domain of Ddx56 was required for the interaction with Sox2. Interestingly, both Ddx56 ΔATP and Ddx56 ΔC-ter truncations could interact with Sox2 by GST pulldown compared with Ddx56 WT (Fig. 4c, d). To further verify the interaction between Ddx56 ΔC-ter and Sox2, we purified His-tagged Sox2 protein from prokaryotic cells and His pulldown was performed using Ddx56 and Ddx56 ΔC-ter expressing cell lysate. The interaction between Ddx56 ΔC-ter and Sox2 was much weaker than the interaction of Ddx56 WT and Sox2 in vitro (Fig. 4f), suggesting loss of helicase C-terminal might decrease the interaction with Oct4/Sox2 complex and be the reason for Ddx56 ΔC-ter decreasing proliferation in mESCs.

## Discussion

**Fig. 5:**
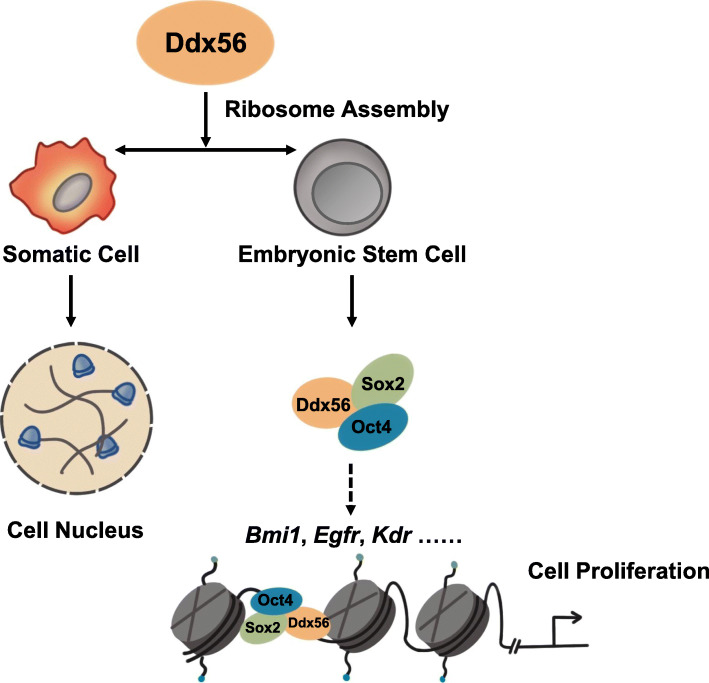
Ddx56 regulates ribosome assembly and maintains the proliferation of mouse embryonic stem cells via interaction with the Oct4/Sox2 complex

It has been difficult to study the function of essential factors in stem cell maintenance, such as nucleolus localizing proteins which are involved in cell growth and survival [13, 18]. In this study, we confirmed that nucleolus localization of Ddx56 regulated ribosome assembly by loss-of-function assays. To identify ribosome-independent and stem cell-specific functions of Ddx56, we found that its helicase C-terminal domain was important in maintaining stem cell proliferation. Deletion of helicase C-terminal domain of Ddx56 did not affect ribosome assembly but decreased the interaction with the Oct4/Sox2 complex, resulting in ESC proliferation delay (Fig. 5).

RNA helicases are multifunctional proteins. Many of them participate in ribosome assembly, but they also have additional functions. For example, Ddx46 inhibits innate immunity [30] and Ddx5 inhibits reprogramming to pluripotency [31]. RNA helicases can also control miRNA biogenesis, thus repressing cell differentiation [23, 32–36]. Whether Ddx56 participates in the post-transcriptional regulation of these genes in mESCs needs further investigation. Herein, based on the results that Ddx56 binds to the Oct4/Sox2 complex, we only focus on its specific function in the pluripotency maintenance of ESCs.

The Oct4/Sox2 complex and its related proteins are crucial for maintaining the proliferation and pluripotency in stem cells [8, 37]. Ddx56 was shown to interact with the Oct4/Sox2 complex. Moreover, we found that the Ddx56 and Sox2 interaction was much stronger than the interaction of Ddx56 and Oct4, compared with Sox2 and Oct4 interaction. Unexpectedly, Ddx56 ΔC-ter mutant showed a defective mESC phenotype of smaller clones and weaker interaction with Sox2. This result suggested that the C-terminal of Ddx56 was indispensable for the interaction with the Oct4/Sox2 complex. It was well known that Oct4 was the core partner for the Oct4/Sox2 complex [8], while the SOX2 activity is regulated by its binding partners, such as SOX2-ZEB1 interaction decreased SOX2 activity and SOX2-FOXA1 interaction increased SOX2 activity [38]. Thus, we hypothesize Ddx56 helicase C-terminal deletion may change the Sox2 interacting pattern and Sox2 activity, thus decreasing ESC proliferation.

Overexpression of Ddx56 ΔC-ter mutant downregulated several stem cell self-renewal-related genes, including *Bmi1*, *Egfr*, *Kdr*. *Bmi1*, and *Egfr*. Specifically, *Bmi1* deficiency in neural stem cells and hematopoietic stem cells decreased self-renewal ability [39, 40]. When knocking down *Egfr* in mESCs by inhibitors or siRNAs, delay in cell proliferation was observed, and the cells would be arrested in the G0/G1 phase [41]. It was not clear whether *Bmi1*, *Egfr*, and *Kdr* were Sox2 target genes. In addition, knockdown of Ddx56 suspended cell cycle progression from G2M to G1 phase in colorectal cancer cell line [42]. These data were consistent in Ddx56 ΔC-ter expressing mESCs in our study.

Ddx56 truncation (the C-terminal deletion) significantly decreased the size of stem cell clones, indicating cell proliferation inhibition and an alteration of cell cycle. It has been reported that cell cycle-related genes control stem cell pluripotency and early differentiation [43, 44]. As core transcription factors, Sox2 and Oct4 bound mitotic chromatin of stem cells, regulating stem cell maintenance at mitosis-to-G1 (M-G1) transition [45]. In our study, we found that Ddx56 truncation (Ddx56 ΔC-ter) decreased the interaction with Sox2, suggesting loss of helicase C-terminal might be the reason for Ddx56 ΔC-ter-induced proliferation inhibition in stem cells.

## Conclusions

Ddx56 maintains ESC proliferation by conventional regulation of ribosome assembly and interaction with the Oct4/Sox2 complex.

## Supplementary information

**Additional file 1 **: **Table S1.** Information of PCR and qRT-PCR primers.

**Additional file 2 **: **Figure S1.***Ddx56* gRNA designing and detection by T7E1. **(A)** Schematic representation of gRNA target sites in Ddx56 protein domain. **(B)** T7E1 assay to examine the cleavage efficiency of gRNA in E14 cells. The star indicates the predicted digested band. **Figure S2.** Ddx56 knockdown affects mESCs survival. **(A)** E14 cells were transfected with control oligos (NC) or three different oligos targeting Ddx56 (si-1, si-2. si-3). After 48 h, cells were collected, and knockdown efficiency was detected by RT–qPCR. Blank, untreated E14 cells. (**B)** E14 cells were transfected with control oligos (NC) or three different oligos targeting Ddx56 (si-1, si-2. si-3). After 48 h, cells were visualized by phase-contrast microscopy. 4×, objective. (left) and analyzed by quantitative real-time polymerase chain reaction (qRT-PCR) to assess knockdown efficiency (right). Blank, untreated E14 cells. si-1A and si-1B mean two parall holes. **(C)** Cells from (A) were analyzed by qRT-PCR for the expression of pluripotency markers and the indicated lineages marker. Error bars indicated SD (n=3), *, p<0.05, ***, p<0.001. Abbreviation: Pre, primitive endoderm. **Figure S3.** Ddx56 wildtype and truncations expression do not affect cell size. **(A)** Western blotting was performed to detect exogenous expression of flag tagged Ddx56 full length or Ddx56 domain truncations in mESCs after the induction of Dox for 2 days. Tubulin or GAPDH served as a loading control. Abbreviation: iOE, inducible overexpression. **(B)** Cells were cultured with (+Dox) or without doxycycline (-Dox) for 4 days, then seeded into a new plate in single cells. 50 cells were calculated in each group. **Figure S4.** Wildtype Ddx56 or Ddx56 ΔC-ter expressing mESCs do not affect the level of pluripotency factors and pluripotency related cell cycle genes. RT-PCR analysis was carried out to detect the pluripotency genes (*Oct4, Nanog, Sox2, Klf4*) and regulating stem cell cycle genes (*Foxd3, Pura, Wnt3a, Myc*) in wildtype Ddx56 or Ddx56 ΔC-ter expressing mESCs. **Figure S5.** Ddx56 is localized in nucleolus with Oct4 in the overexpressed cells. EGFP or Ddx56 induced expression mESCs were cultured with or without Dox for 2 days, and subjected to immunostaining with antibodies against Oct4 (green) and Flag (red).

## Data Availability

All data generated and/or analyzed in this study are included in this published article.

## Abbreviations

ESCs: Embryonic stem cells
mESCs: Mouse embryonic stem cells
IP-MS: Immunoprecipitation and mass spectrometry
Npm1: Nucleophosmin 1
Cre: Cyclization recombination enzyme
Ddx56: DEAD-box helicase 56
WT: Wildtype
iCas9 mESCs: Inducible expressing Cas9 in A17-2loxP mESCs
iKO mESCs: Inducible knockout mESCs
Ddx56 iOE mESCs: Ddx56 inducible overexpression mESCs
Ddx56 ΔATP: Helicase ATP binding domain deletion of Ddx56
Ddx56 ΔC-ter: Helicase C-terminal domain deletion of Ddx56
Ddx56 ΔATP iOE mESCs: Ddx56 ΔATP inducible overexpression mESCs
Ddx56 ΔC-ter iOE mESCs: Ddx56 ΔC-ter inducible overexpression mESCs
siRNA: Small interfering RNA
FACS: Fluorescence-activated cell sorting
co-IP: Co-immunoprecipitation
T7EI: T7 endonuclease I
GO analysis: Gene Ontology analysis
DEGs: Differentially expressed genes
