# Supplementary material for: Ddx56 maintains proliferation of mouse embryonic stem cells via ribosome assembly and interaction with the Oct4/Sox2 complex
Source: Stem Cell Res Ther. 2020 Jul 23;11:314. doi: 10.1186/s13287-020-01800-w (PMC7376950; doi:10.1186/s13287-020-01800-w)
Supplement: Supplementary file 1 — Additional file 1 : Table S1. Information of PCR and qRT-PCR primers. [file 13287_2020_1800_MOESM1_ESM.pdf]

Additional file 1 Table S1 Information of PCR and qRT-PCR primers.	
Ddx56-gRNA1-T7E1-FP	CGGAGGGCGTTCAAGGGACAGCATT
Ddx56-gRNA1-T7E1-RP	TTGGCACAGTCGGGCACTTCATTCC
Ddx56-gRNA2-T7E1-FP	TGTAGTTGCTTTGGTTAGTGAGGCAGTGTCC
Ddx56-gRNA2-T7E1-RP	GAGGCGGGAGGAGAAGAAATAAGACCAG
Ddx56-qPCR-FP	GCCAACAACCCAGGCATAGT
Ddx56-qPCR-RP	TGGTATGGAAGCAGGATGGG
Oct4-qPCR-FP	GCAGGAGCACGAGTGGAAAGCAAC
Oct4-qPCR-RP	CAAGGCCTCGAAGCGACAGATG
Nanog-qPCR-FP	AGGCTTTGGAGACAGTGAGGTGC
Nanog-qPCR-RP	TACCCTCAAACTCCTGGTCCTTC
Sox2-qPCR-FP	CGAGATAAACATGGCAATCAAATG
Sox2-qPCR-RP	AACGTTTGCCTTAAACAAGACCAC
mGAPDH-qPCR-FP	TCCCACTCTTCCACCTTCGATGC
mGAPDH-qPCR-RP	GGGTCTGGGATGGAAATTGTGAGG
Gata4-qPCR-FP	TTCCTGCTCGGACTTGGGAC
Gata4-qPCR-RP	TTCCCAGGCAGGTGGAGAATAAG
Gata6-qPCR-FP	ACAGCCCACTTCTGTGTTCCC
Gata6-qPCR-RP	GTGGGTTGGTCACGTGGTACAG
Sox7-qPCR-FP	ATGCTGGGAAAGTCATGGAAG
Sox7-qPCR-RP	CGTGTTCTGGTCACGAGAGA
Foxa2-qPCR-FP	AGCACCATTACGCCTTCAAC
Foxa2-qPCR-RP	CCTTGAGGTCCATTTTGTGG
Sox17-qPCR-FP	AAGAAACCCTAAACACAAACAGCG
Sox17-qPCR-RP	TTTGTGGGAAGTGGGATCAAGAC
Brachyury-qPCR-FP	CTCTAATGTCCTCCCTTGTTGCC
Brachyury-qPCR-RP	TGCAGATTGTCTTTGGCTACTTTG
Nestin-qPCR-FP	CTGCAGGCCACTGAAAAGTTC
Nestin-qPCR-RP	TCTGACTCTGTAGACCCTGCTTCTC
Mash1-qPCR-FP	GCCACCAGAATGACTTCAGCAC
Mash1-qPCR-RP	AAGGCAACCTATGGGAACCAAC
Gpc3-qPCR-FP	CAGCCCGGACTCAAATGGG
Gpc3-qPCR-RP	CAGCCGTGCTGTTAGTTGGTA
Fgf10-qPCR-FP	TTTGGTGTCTTCGTTCCCTGT
Fgf10-qPCR-RP	TAGCTCCGCACATGCCTTC
Mt3-qPCR-FP	CTGGATATGGACCCTGAGACCT
Mt3-qPCR-RP	CTTCACCTTTGCACACACAGTC
Chrnb2-qPCR-FP	AGGGGTTTTGGGTACTGACAC
Chrnb2-qPCR-RP	AGCTTGTTATAGCGGGAAGGA
Klf5-qPCR-FP	TGGAGAAGCGACGTATCCAC
Klf5-qPCR-RP	AGGTGCACTTGTAGGGCTTC
CD38-qPCR-FP	TCTTGCCCACATTGGAGTGAA
CD38-qPCR-RP	ACCACACCACAGGCATCTTC
Tgfbr3-qPCR-FP	TACACTGGGCCCAAAAGGAA
Tgfbr3-qPCR-RP	TACACGTGGGAGGGAACACT
Bmi1-qPCR-FP	TTGTACGCGAGAACATACAGC
Bmi1-qPCR-RP	CTGAGTCGGGTCCTTTGGC
Egfr-qPCR-FP	GCCATCTGGGCCAAAGATACC
Egfr-qPCR-RP	GTCTTCGCATGAATAGGCCAAT
Kctd11-qPCR-FP	CTGGGGGCCATGTTTAGGG
Kctd11-qPCR-RP	AATTGAGGATGTGCCGGAAAG
Kdr-qPCR-FP	TTTGGCAAATACAACCCTTCAGA
Kdr-qPCR-RP	GCAGAAGATACTGTCACCACC
Ford3-qPCR-FP	GACCCCGAACAAGCCCAAG
Ford3-qPCR-RP	GAAAACGGTTGCTGATGAACTC
Wnt3a-qPCR-FP	CTCCTCTCGGATACCTCTTAGTG
Wnt3a-qPCR-RP	CCAAGGACCACCAGATCGG
Pura-qPCR-FP	CATCGACGACTATGGAGTGGA

Pura-qPCR-RP	TGTTGGAACCCACATCGAAGA
