# Supplementary material for: Ddx56 maintains proliferation of mouse embryonic stem cells via ribosome assembly and interaction with the Oct4/Sox2 complex
Source: Stem Cell Res Ther. 2020 Jul 23;11:314. doi: 10.1186/s13287-020-01800-w (PMC7376950; doi:10.1186/s13287-020-01800-w)

Additional file 2 Figure S1

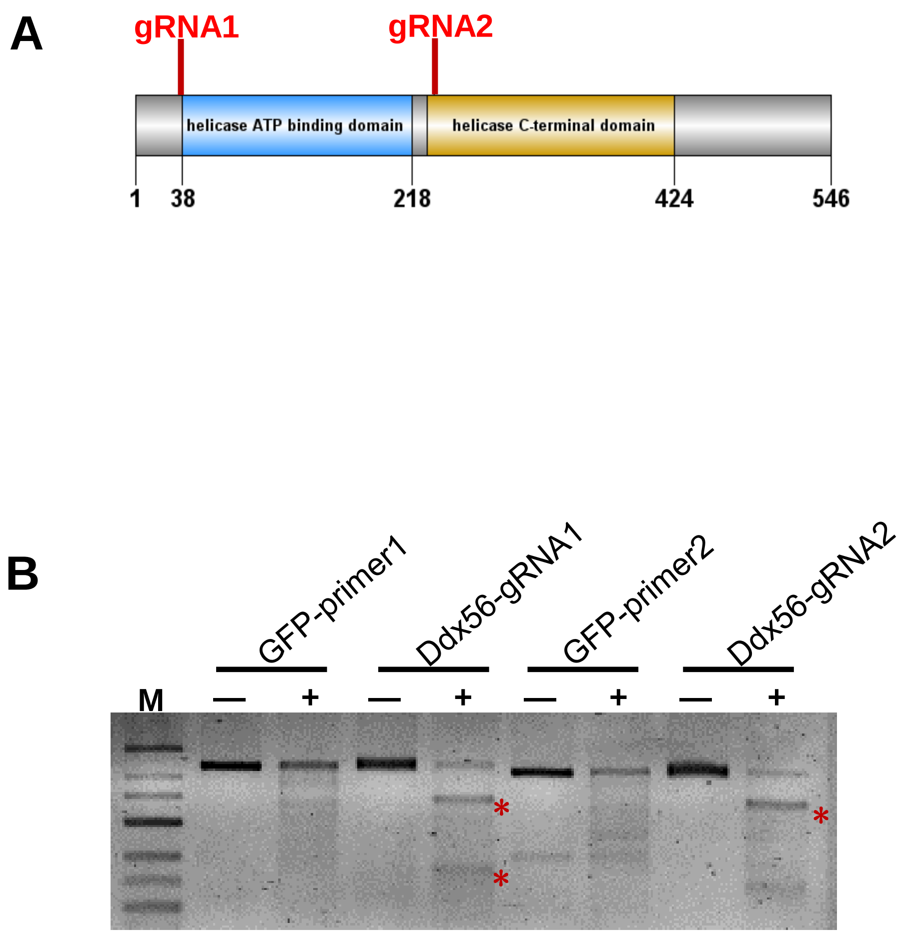

Additional file 2 Figure S2

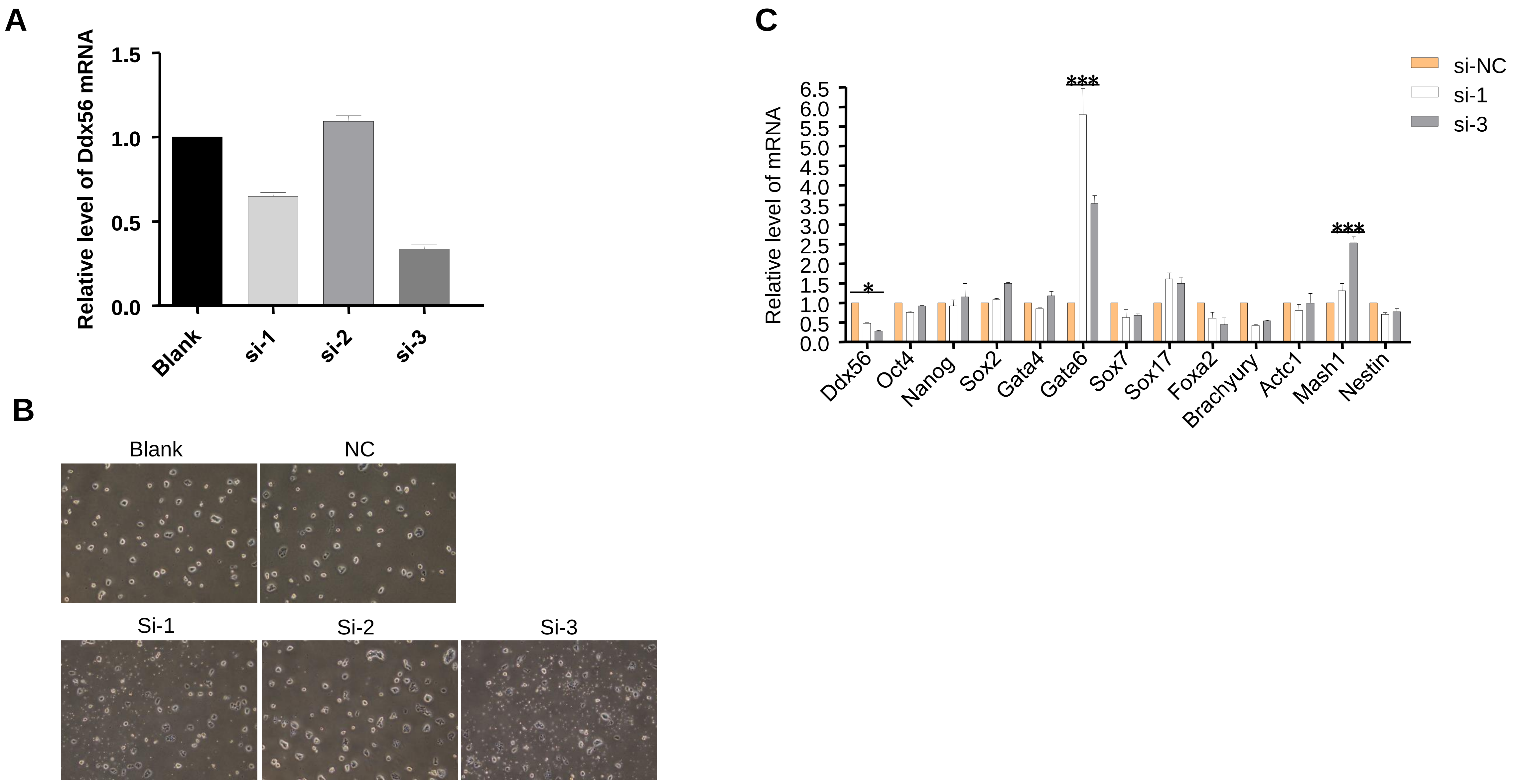

Additional file 2 Figure S3

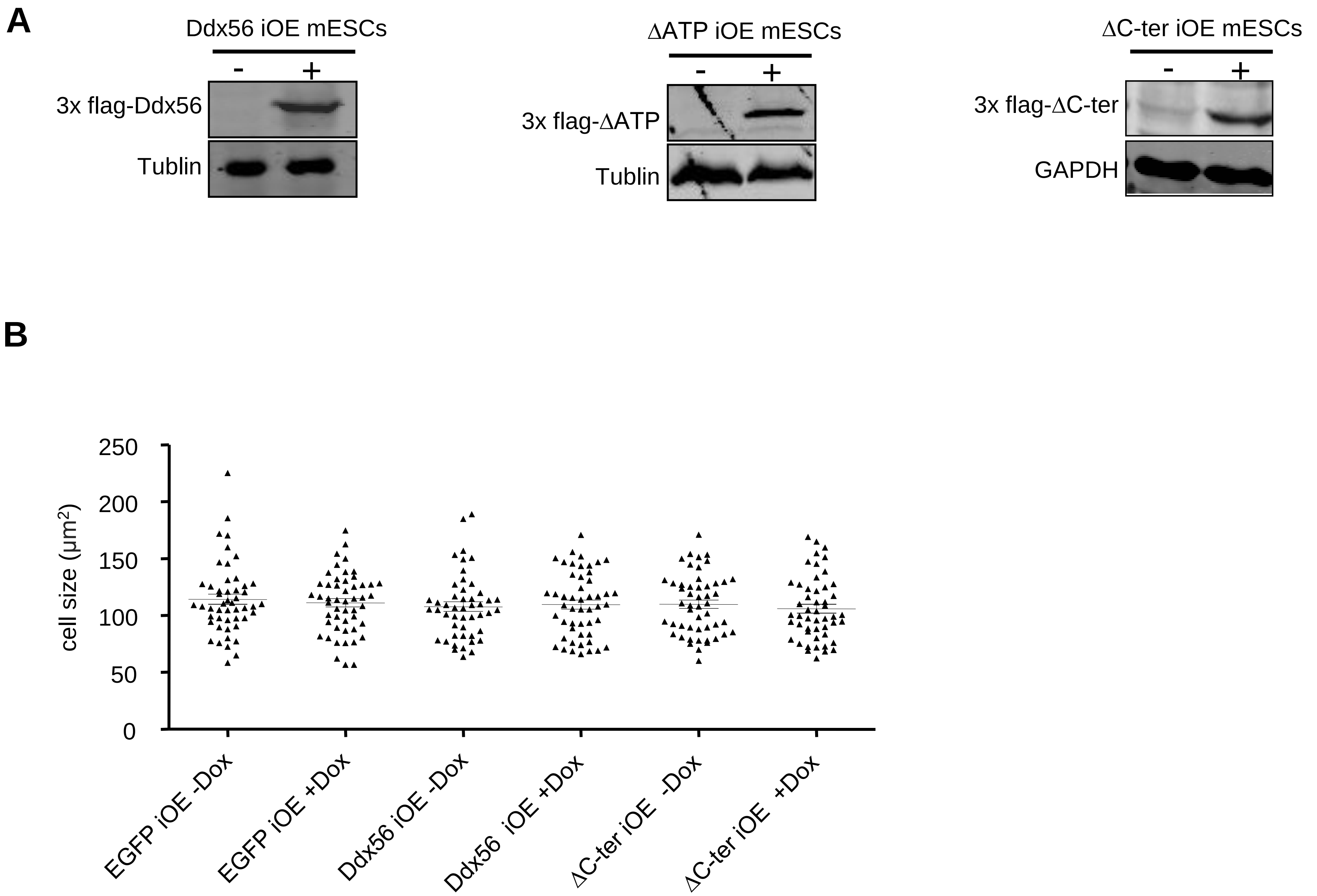

Additional file 2 Figure S4

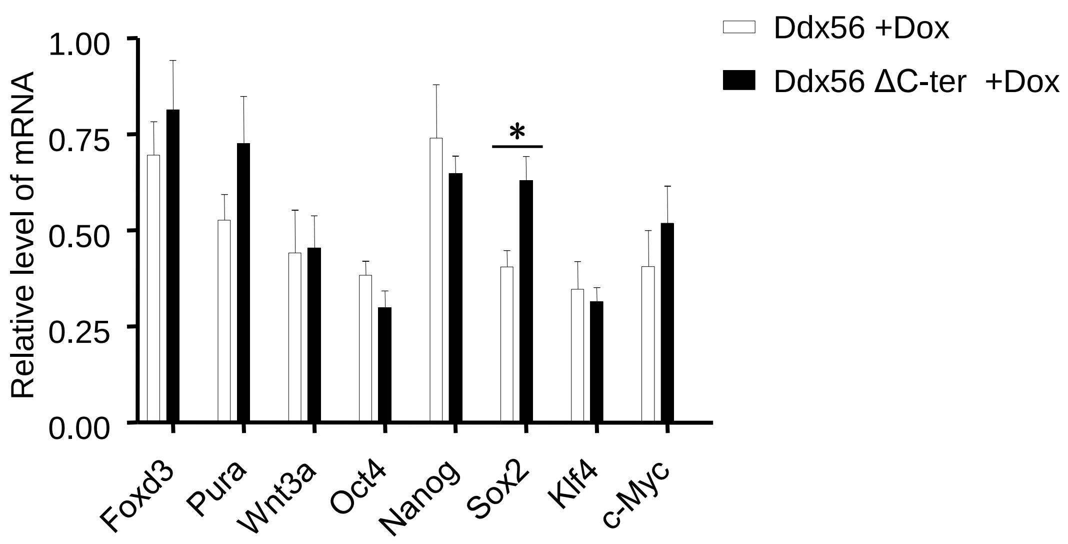

Additional file 2 Figure S5

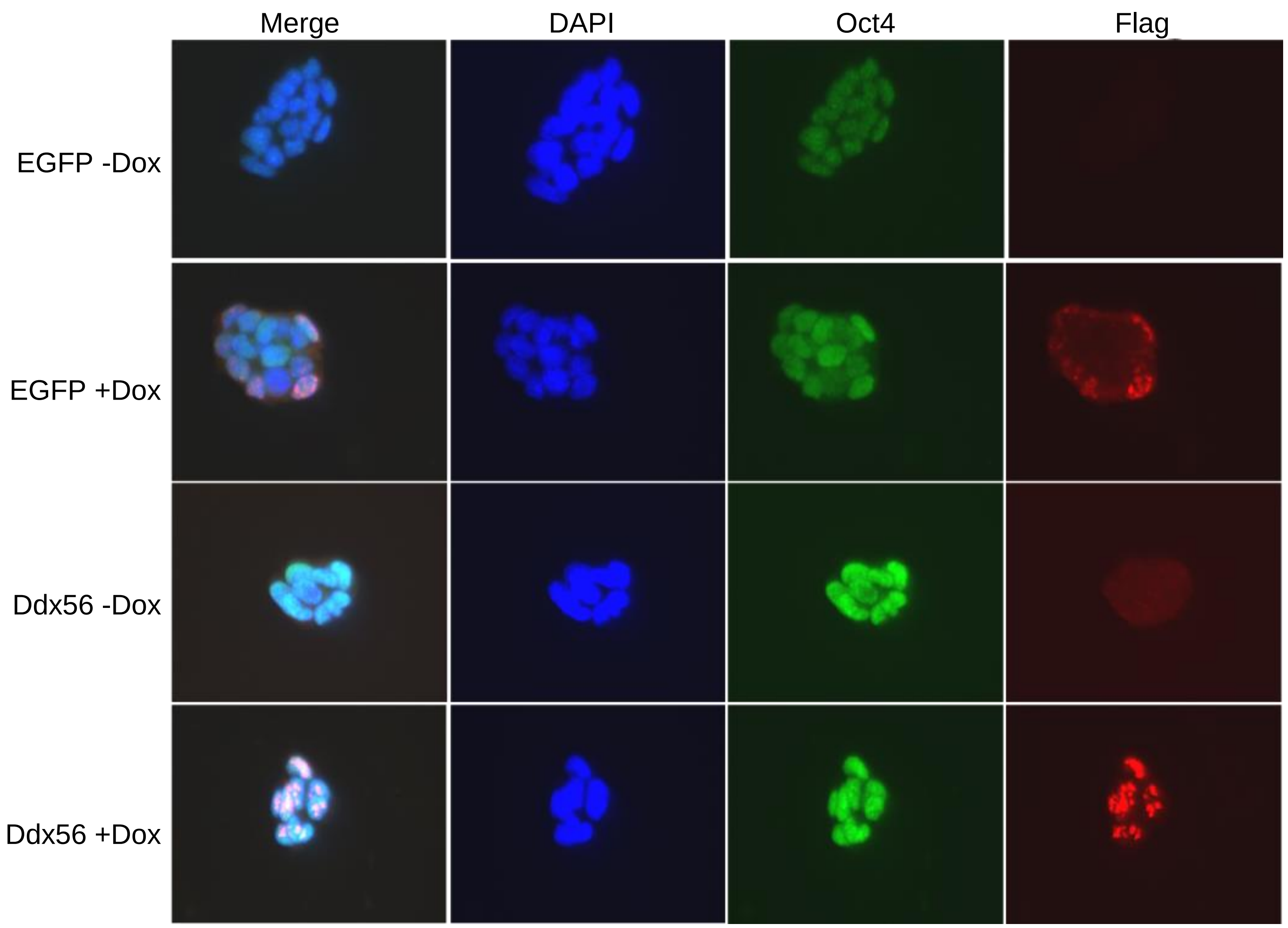

Supplement: Supplementary file 2 — Additional file 2 : Figure S1.Ddx56 gRNA designing and detection by T7E1. (A) Schematic representation of gRNA target sites in Ddx56 protein domain. (B) T7E1 assay to examine the cleavage efficiency of gRNA in E14 cells. The star indicates the predicted digested band. Figure S2. Ddx56 knockdown affects mESCs survival. (A) E14 cells were transfected with control oligos (NC) or three different oligos targeting Ddx56 (si-1, si-2. si-3). After 48 h, cells were collected, and knockdown efficiency was detected by RT–qPCR. Blank, untreated E14 cells. (B) E14 cells were transfected with control oligos (NC) or three different oligos targeting Ddx56 (si-1, si-2. si-3). After 48 h, cells were visualized by phase-contrast microscopy. 4×, objective. (left) and analyzed by quantitative real-time polymerase chain reaction (qRT-PCR) to assess knockdown efficiency (right). Blank, untreated E14 cells. si-1A and si-1B mean two parall holes. (C) Cells from (A) were analyzed by qRT-PCR for the expression of pluripotency markers and the indicated lineages marker. Error bars indicated SD (n=3), *, p<0.05, ***, p<0.001. Abbreviation: Pre, primitive endoderm. Figure S3. Ddx56 wildtype and truncations expression do not affect cell size. (A) Western blotting was performed to detect exogenous expression of flag tagged Ddx56 full length or Ddx56 domain truncations in mESCs after the induction of Dox for 2 days. Tubulin or GAPDH served as a loading control. Abbreviation: iOE, inducible overexpression. (B) Cells were cultured with (+Dox) or without doxycycline (-Dox) for 4 days, then seeded into a new plate in single cells. 50 cells were calculated in each group. Figure S4. Wildtype Ddx56 or Ddx56 ΔC-ter expressing mESCs do not affect the level of pluripotency factors and pluripotency related cell cycle genes. RT-PCR analysis was carried out to detect the pluripotency genes (Oct4, Nanog, Sox2, Klf4) and regulating stem cell cycle genes (Foxd3, Pura, Wnt3a, Myc) in wildtype Ddx56 or Ddx56 ΔC-ter e [file 13287_2020_1800_MOESM2_ESM.pdf]
